# Introducing a pharmacist-led transmural care program to reduce drug-related problems in orthogeriatric patients: a prospective interventional study

**DOI:** 10.1186/s12877-023-04591-w

**Published:** 2024-01-11

**Authors:** Rachel Bailly, Stephanie Wuyts, Loic Toelen, Tony Mets, Carmen Van Hauwermeiren, Thierry Scheerlinck, Pieter-Jan Cortoos, Siddhartha Lieten

**Affiliations:** 1 Departement of Pharmacy, Universitair Ziekenhuis Brussel (UZ Brussel), Brussels, Belgium; 2 Research Group Clinical Pharmacology and Clinical Pharmacy, Faculty of Medicine and Pharmacy, Vrije Universiteit Brussel, Brussels, Belgium; 3 Faculty of Medicine and Pharmacy, Vrije Universiteit Brussel, Brussels, Belgium; 4 Department of Geriatrics, Universitair Ziekenhuis Brussel (UZ Brussel), Brussels, Belgium; 5 Department of Orthopedics and Traumatology, Universitair Ziekenhuis Brussel (UZ Brussel), Brussels, Belgium

**Keywords:** Interventional study, Orthogeriatric patients, Multidisciplinary approach, Clinical pharmacist, Medication review, Drug-related problems, Transmural care program

## Abstract

**Background:**

Orthogeriatric patients have an increased risk for complications due to underlying comorbidities, chronic drug therapy and frequent treatment changes during hospitalization. The clinical pharmacist (CP) plays a key role in transmural communication concerning polypharmacy to improve continuity of care by the general practitioner (GP) after discharge. In this study, a pharmacist-led transmural care program, tailored to orthogeriatric patients, was evaluated to reduce drug related problems (DRPs) after discharge.

**Methods:**

An interventional study was performed (pre-period: 1/10/2021-31/12/2021; post-period: 1/01/2022-31/03/2022). Patients (≥ 65 years) from the orthopedic department were included. The pre-group received usual care, the post-group received the pharmacist-led transmural care program. The DRP reduction rate one month after discharge was calculated. Associated factors for the DRP reduction rate were determined in a multiple linear regression analysis. The GP acceptance rate was determined for the proposed interventions, as well as their clinical impact using the Clinical, Economic and Organizational (CLEO) tool. Readmissions one month after discharge were evaluated.

**Results:**

Overall, 127 patients were included (control *n* = 61, intervention *n* = 66). The DRP reduction rate was statistically significantly higher in the intervention group compared to the control group (*p < 0.001*). The pharmacist’s intervention was associated with an increased DRP reduction rate (+ 1.750, 95% confidence interval 1.222–2.278). In total, 141 interventions were suggested by the CP, of which 71% were accepted one month after discharge. In both periods, four patients were readmitted one month after discharge. 58% of the interventions had a clinical impact (≥ 2 C level using the CLEO-tool) according to the geriatrician and for the CP it was 45%, indicating that they had the potential to avoid patient harm.

**Conclusions:**

The pharmacist-led transmural care program significantly reduced DRPs in geriatric patients from the orthopedic department one month after discharge. The transmural communication with GPs resulted in a high acceptance rate of the proposed interventions.

## Key messages

Orthogeriatric patients are at increased risk for developing DRPs due to underlying comorbidities, polypharmacy, treatment changes during hospitalization, and limited expertise on complex pharmacotherapy among most surgeons. Several studies have shown a positive effect of CP interventions and multidisciplinary approaches on reducing DRPs.

Even though a lot of studies recognize the importance of interdisciplinary collaboration to implement drug-specific recommendations, only a few have included the GP at discharge to discuss hospital-based recommendations.

This study showed that a multifaceted pharmacist-led intervention with a primary care directed approach, proved to be effective in order to resolve DRPs and to ensure continuity of care after discharge. In future studies, the patient and community pharmacist should be actively involved to further reduce DRPs.

## Introduction

Older patients frequently suffer from multiple comorbidities, increasing the risk of polypharmacy and therefore drug-related problems (DRPs) [1]. According to pharmaceutical care network of Europe (PCNE), *DRP is defined as, “an event or circumstance involving drug therapy that actually or potentially interferes with desired health outcomes”* [2]. Fall-related injuries are a major health issue in older people, as they are not only associated with additional rehabilitation, medical, and social complications, but also with a significant economic burden on the health care systems [3]. When geriatric patients with fall-associated injuries are admitted for surgical treatment, their risk for complications is increased due to underlying comorbidities, polypharmacy, frequent treatment changes during hospitalization, and limited expertise on complex pharmacotherapy among most surgeons. As such, geriatric counseling is recommended for older patients on an orthopedic ward [4, 5].

Several studies have demonstrated that a multidisciplinary approach can improve outcomes in terms of hospital readmissions, and quality of life (QOL), of these patients [2, 6]. An orthogeriatric co-management (OG-CM) model is a sophisticated model for the management of frail patients in which a geriatrician is integrated into the orthopedic ward, to manage the patient together with the orthopedic surgeon from admission to discharge [7]. This model demonstrates an increase in quality of care, as evidenced by the increased number of diagnoses of comorbidities resulting in having less readmissions, which is beneficial for health care systems [7]. Besides the geriatrician, the clinical pharmacist (CP) can help to optimize pharmacotherapy [2], by means of medication reconciliation (MR), review, identifying fall-risk increasing drugs (FRIDs), counseling of the patient or caregiver and post-discharge follow-up [8, 9]. Transmural communication to primary care providers (PCPs) such as general practitioners and community pharmacists is essential, especially at crucial moments, such as care transitions [10]. There is a growing body of literature that recognizes the importance of interdisciplinary collaboration to implement drug-specific recommendations. The general practitioner (GP), for example, plays a key role as he maintains an overview of all the patient’s prescribed medication and comorbidities. However, only a few studies have included the GP at discharge to discuss hospital-based recommendations [2].

Therefore, the aim of this study was to investigate the impact of pharmaceutical interventions (PIs) with regard to the DRP reduction rate after discharge, in geriatric patients admitted to the orthopedic ward.

## Methods

A prospective, monocentric interventional study with a pre-post design was conducted on the orthopedic ward (29 beds) of the University Hospital of Brussels, a 721-bed tertiary hospital in Belgium. Patients in the pre-group received usual care including the OG-CM model (October 1st to December 31st 2021), while patients in the post-group received the multidisciplinary approach including pharmacist-led interventions (January 1st to March 31st 2022). A pre-post approach was chosen to prevent contamination bias in the usual care group.

### Study population

Inclusion criteria were patients aged 65 or more, patients with an orthopedic problem admitted to orthopedic or other surgical wards, either through the emergency department or after ambulatory specialist referral, hospitalization for > 48 h, and Dutch or French speaking. Exclusion criteria were logistical reasons (e.g., isolation due to COVID), refusal of informed consent and a setting of palliative care.

### Study procedure

During the control period, usual care concerning drug therapy of the included patients was documented; no PIs were carried out. The ward’s residents were responsible for MR, review and pharmaceutical follow-up at discharge. There was a full-time geriatrician collaborating with surgeons (OG-CM model) who focused on older patients as part of an integrated co-management strategy (orthopedic and trauma surgeons, a member of the Geriatric Liaison Service, a dietician, a physical therapist and a social nurse). According local policy, drug-related recommendations were preferably made upon discharge due to the patient’s short length of stay (LOS). Treatment changes only occurred if deemed necessary by the geriatrician.

In the intervention period, a CP was added to the OG-CM model. The pharmacist-led intervention included MR (conducted with the help of PCPs such as the community pharmacist (CoP) and GP), medication review during hospitalization, optimizing patient counseling at discharge and post-discharge follow-up of patients. At discharge, the identified DRPs were discussed with both the geriatrician and surgical resident to determine which interventions could be proposed to the GP in the discharge letter. The CP provided a transitional pharmaceutical care plan which was integrated in the discharge letter, as well as patient counseling [11]. At the end of the hospitalization, the CP reviewed the medication for remaining (potentially avoidable) DRPs. This was done using explicit medication assessment tools (GheOP^3^S tool (Ghent Older People’s Prescriptions community Pharmacy Screening) [12] and Stockley’s Interactions Checker [13]). The CP contacted the GP at discharge to discuss changes and he proposed a follow-up plan for drug-related interventions.

One month after discharge, the patient’s GP was contacted again to reevaluate the home medication and to determine unresolved DRPs. If the GP could not be reached, the current medication was obtained by contacting the CoP.

Patient characteristics (age, gender, type of residence before admission, reason for admission, Charlson Comorbidity Index (CCI) [14], medication fall-risk score [15]) and data concerning the hospitalization were collected from the electronic patient records in the hospital’s information system (PrimUZ®). Medication-related information (MR, data concerning patient counseling and follow-up) was also documented during both periods. All data were registered in an online database on the REDCap® platform (Vanderbilt University, Nashville, TN, USA).

### Outcomes

The DRP reduction rate was calculated and compared in the control- and intervention-group as the primary outcome, by determining the difference between the amount of DRPs at discharge and the amount one month later.

Additionally, GP acceptance rates were determined for the proposed PIs in the intervention-group, differentiating between interventions accepted immediately at discharge and those accepted after one month. Readmissions within one month following discharge were documented. An estimation of the clinical impact (CI) of the proposed PIs, was done using the methodology as proposed in the Clinical, Economic and Organizational (CLEO) tool [16]. All the PIs were scored by an independent geriatrician and CP. Clinically significant PIs were those with a CI ≥ 2 C [16].

### Data analysis

Data are presented as means and standard deviation (SD) or median with interquartile range (IQR) as appropriate. Frequencies (percentage) were calculated for categorical variables. The unpaired *t*-test and Mann-Whitney U-test were used to compare continuous variables and the Chi-square test for frequencies with Bonferroni correction where needed. The impact of the intervention on DRP reduction rate, as well as other patient- or drug-related characteristics was explored in a stepwise multiple linear regression analysis. The final model was tested for multicollinearity, homoscedasticity and normality of residuals. *P-*values less than 0.05 (two- sided) were considered statistically significant. A weighted Cohen’s Kappa (κ_w_) analysis was done to determine the inter-rater agreement between the two healthcare providers scoring the CI of PIs [17]. All data were analyzed using IBM SPSS Statistics® version 28.0 (IBM Corporation, Armonk, NY, USA).

## Results

### Baseline patient characteristics

**Fig. 1:**
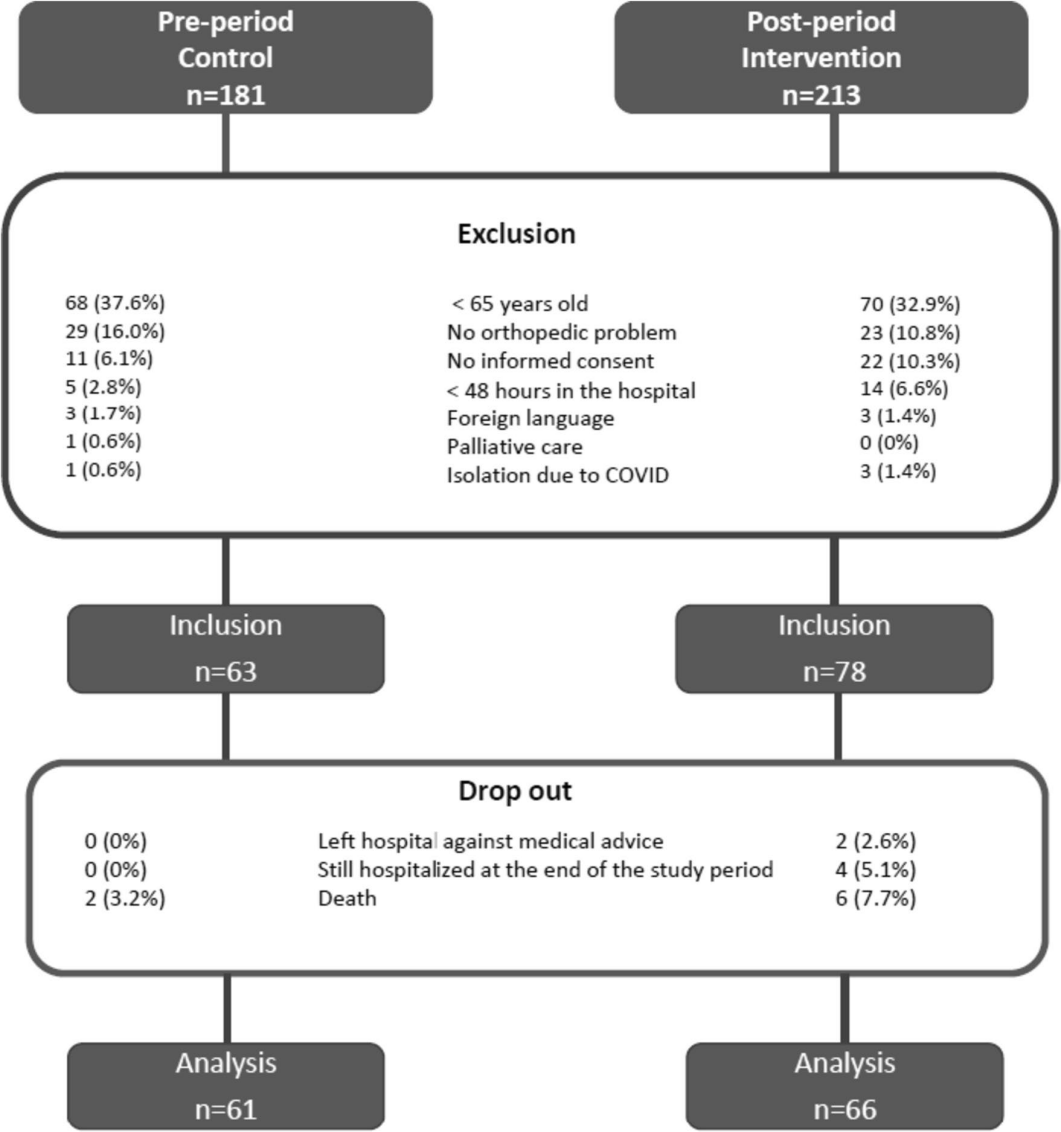
Description of the patient inclusion process

Of 394 eligible patients, 141 (36%) patients were included (Fig. 1). The main exclusion reason was age < 65 years (35%). Fourteen patients did not complete the study because of death (*n* = 8), still hospitalized at the end of the study period (*n* = 4), or left the hospital against medical advice (*n* = 2). A total of 127 patients were included (control = 61 patients; intervention = 66 patients).

The baseline patient characteristics of both groups were similar (Table 1). However, intervention patients had a longer LOS (11 days vs. 7 days; *p* = 0.013).

**Table 1:** Baseline patient characteristics

	**Control** **(*n* = 61)**	**Intervention** **(*n* = 66)**	***p*-value**
Age (years); (mean, SD^a^)	79 (7.7)	80 (8.4)	0.599^d^
Gender (male); (n,%)	18 (29.5)	16 (24.2)	0.503^e^
***Housing before admission; (n,%)***			0.109^e^
Home	55 (90.2)	51 (77.3)	
Nursing home	4 (6.6)	13 (19.7)	
Serviceflat	1 (1.6)	0 (0)	
Rehabilitation facility	1 (1.6)	2 (3.0)	
Recent hospitalization; (n,%)	6 (9.8)	13 (19.7)	0.120^e^
***Type of admission; (n,%)***			0.086^e^
Urgent	24 (39.3)	36 (54.5)	
Elective	37 (60.7)	30 (45.5)	
***Diagnosis on admission; (n,%)***			0.121^e^
Hip and lower limb problems	54 (88.5)	49 (74.2)	
Upper limb problems	5 (8.2)	12 (18.2)	
Spinal problems	2 (3.3)	5 (7.6)	
**Quality of care parameters**			
CCI^b^; (median, IQR^c^)	4 (2)	5 (3)	0.075^f^
Fall-risk score; (median, IQR)	6 (5)	5.5 (6)	0.990^f^
Patient underwent surgery; (n,%)	57 (93.4)	61 (92.4)	0.823^e^
***Discharged to; (n,%)***			0.108^e^
Home	40 (65.6)	34 (51.5)	
Rehabilitation facility	21 (34.4)	32 (48.5)	
LOS; (median, IQR)	7 (8)	11 (12)	**0.013**^f^
Number of drugs on admission per patient; (median, IQR)	6 (8)	8 (5)	**0.016**^f^
Number of drugs at discharge per patient; (median, IQR)	8 (6)	10 (6)	**0.015**^f^
Number of DRPs at discharge per patient; (median, IQR)	3 (4)	3 (4)	0.846^f^
Readmissions one month after discharge; (n,%)	4 (6.6)	4 (6.1)	0.908^e^

^a^*SD* standard deviation; ^b^*CCI* Charlson Comorbidity Index; ^c^*IQR* Interquartile range

Statistical analyses were performed using the ^d^T-test, ^e^Chi square test, ^f^Mann-Whitney U-test

### DRP reductions after pharmacist intervention

Overall, 201 DRPs were detected at discharge during the control period by the CP, 223 DRPs in the intervention period. No DRPs were identified in 6 patients in the control period and 10 in the intervention period. The most common DRPs were potentially inappropriate medication (PIMs) like the combination of FRIDs (15.1%) (Table 2, Part 4), the use of opioids (12.0%), PPIs (proton-pump inhibitors) > 8 weeks (12.0%) and benzodiazepines or Z-drugs (9.2%) (Table 2).

**Table 2:** Detected DRPs in patients during the control- and intervention-period using the GheOP^3^s tool

Drug-Related Problems (DRPs)	Control (*n* = 223)	Control 1 month after discharge (*n* = 178)	Intervention (*n* = 223)	Intervention 1 month after discharge (*n* = 99)
**Part 1: potentially inappropriate drugs, independent of diagnosis**				
Opioids	30 (14.9)	18 (10.1)	21 (9.4)	12 (12.1)
PPI^a^ >8 weeks	19 (9.4)	22 (12.3)	32 (14.3)	14 (14.1)
Benzodiazepines or Z-drugs	18 (9.0)	25 (14.0)	21 (9.4)	16 (16.2)
Acetylsalicylic acid > 100 mg/day	16 (8.0)	3 (1.7)	10 (4.5)	0 (0)
Antidepressants > 1 year	6 (3.0)	7 (3.9)	8 (3.6)	2 (2.0)
Systemic NSAIDs^b^	3 (1.5)	5 (2.8)	6 (2.7)	1 (1.0)
Centrally-acting antihypertensives	2 (1.0)	2 (1.1)	2 (0.9)	1 (1.0)
Alizapride	2 (1.0)	0 (0)	0 (0)	0 (0)
Antipsychotics > 1 month	1 (0.5)	1 (0.6)	5 (2.2)	4 (4.0)
Contact laxatives for daily use > 2 weeks	1 (0.5)	2 (1.1)	5 (2.2)	3 (3.0)
Metoclopramide	1 (0.5)	1 (0.6)	1 (0.4)	0 (0)
Barbiturates	1 (0.5)	1 (0.6)	0 (0)	0 (0)
Oral decongestants	1 (0.5)	2 (1.1)	0 (0)	0 (0)
Long-acting sulphonylurea derivatives	0 (0)	0 (0)	1 (0.4)	0 (0)
Narcotic antitussives	0 (0)	2 (1.1)	0 (0)	0 (0)
Sedating antihistaminic	0 (0)	1 (0.6)	0 (0)	0 (0)
**Part 2: potentially inappropriate drugs, dependent of diagnosis**				
Drugs likely to cause constipation in patients with known constipation	14 (6.9)	11 (6.2)	15 (6.7)	6 (6.1)
Drugs with anticholinergic properties with known dementia/cognitive impairment	6 (3.0)	6 (3.4)	4 (1.8)	2 (2.0)
Systemic predniso(lo)ne-equivalents > 7.5 mg/day with diabetes	1 (0.5)	1 (0.6)	1 (0.4)	1 (1.0)
Drugs with anticholinergic properties with known benign prostatic hyperplasia	1 (0.5)	1 (0.6)	1 (0.4)	0 (0)
**Part 3: Potentially omitted medication in older people**				
Opioids without laxative	18 (8.9)	8 (4.5)	8 (3.6)	0 (0)
Osteoporotic therapy without adequate calcium/vitamin D	6 (3.0)	4 (2.2)	4 (1.8)	1 (1.0)
Predniso(lo)ne-equivalent of ≥ 7.5 mg for ≥ 3 months without calcium/vitamin D supplementation and bisphosphonate	2 (1.0)	1 (0.6)	1 (0.4)	0 (0)
**Part 4: Drug-Drug interactions of specific relevance**				
Combination of FRIDs	29 (14.4)	29 (16.3)	35 (15.7)	21 (21.2)
Combination of drugs with anticholinergic properties	8 (4.0)	7 (3.9)	17 (7.6)	11 (11.1)
Combination of QT prolonging drugs or combination of QT prolonging drug and drug that inhibits metabolism of this drug	6 (3.0)	5 (2.8)	13 (5.8)	4 (4.0)
Combination of drugs leading to increased bleeding risk	5 (2.5)	7 (3.9)	5 (2.2)	0 (0)
RAAS^c^ inhibitor + potassium sparing diuretic/potassium supplement/potassium containing drug	1 (0.5)	1 (0.6)	4 (1.8)	0 (0)
Systemic NSAID + RAAS inhibitor	1 (0.5)	3 (1.7)	3 (1.3)	0 (0)
Oral antidiabetics with risk of hypoglycaemia/insulin + non-selective β-blocker	1 (0.5)	1 (0.6)	0 (0)	0 (0)
Systemic NSAID + Diuretic	1 (0.5)	1 (0.6)	0 (0)	0 (0)

^a^*PPI* proton-pump inhibitors

^b^*NSAIDs* Non-steroidal anti-inflammatory drugs

^c^*RAAS* Renin-angiotensin-aldosteronesystem

**Fig. 2:**
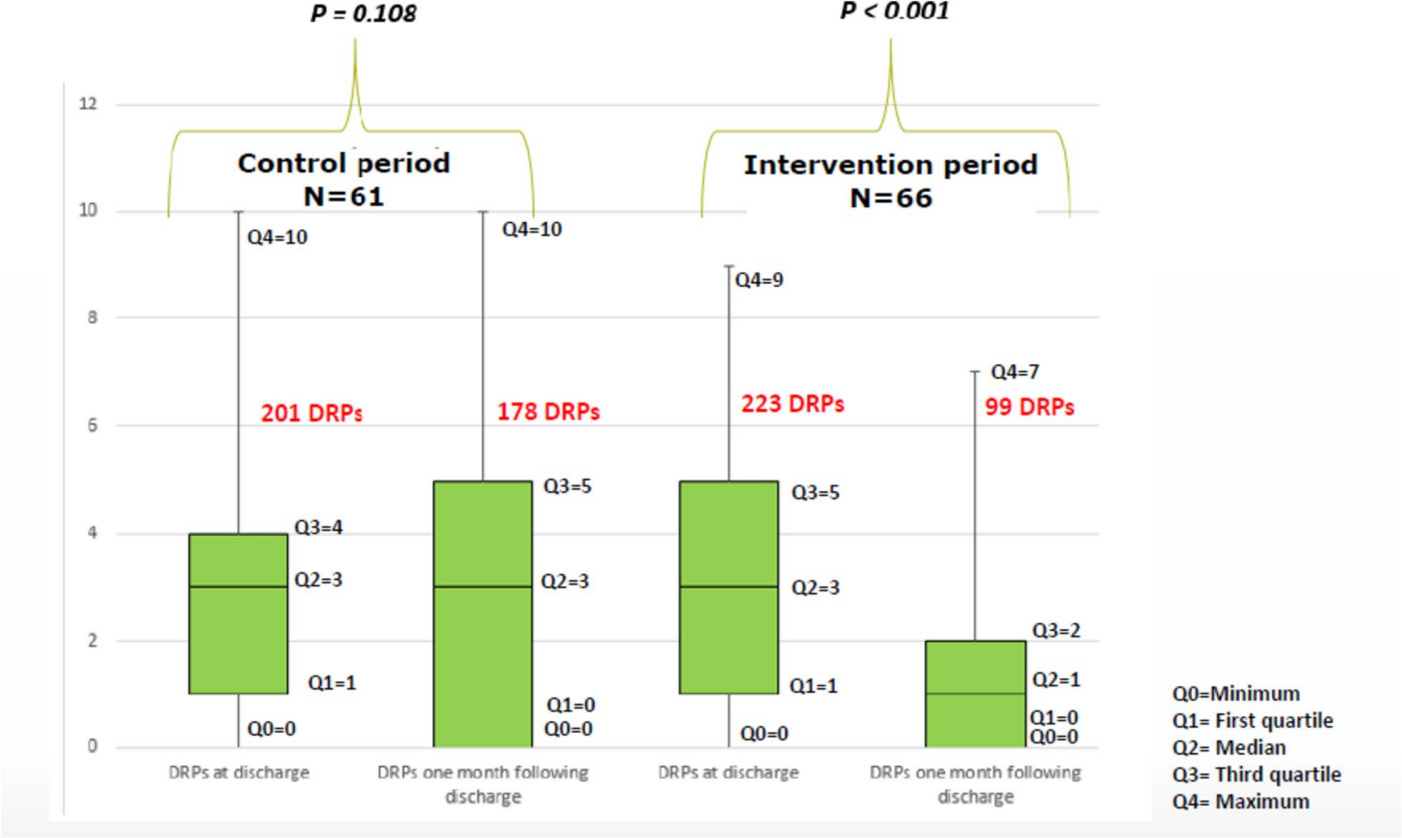
Number of DRPs at discharge and one month following discharge for both periods

The reduction of DRPs in the intervention period (median = 1.0) was significantly higher than in the control period (median = 0.0) (U = 1194, *p* < 0.001). In the usual care group, 178 of the 201 DRPs (88%) were still present one month after discharge versus 99 of the 223 DRPs (44%) for patients receiving the multidisciplinary intervention (Fig. 2).

The multiple linear regression analysis (Table 3) showed that the intervention itself, and number of DRPs at discharge significantly increased the reduction rate for DRPs. In contrast, recent hospitalization, increasing fall-risk score on admission, and the number of drugs on admission led to lower DRP reduction rate. Age, CCI and urgent admission had no significant influence in this model. The residuals in this model were normally distributed and homoscedastic, and no multicollinearity was observed (Variance Inflation Factor values < 5).

**Table 3:** Multiple linear regression analysis

Independent variables	Parameter estimate	95% Confidence Interval	*p*-value
**(Constant)**	-2.918	-5.764 ─ (-0.073)	0.044
**Age**	0.039	0.000 ─ 0.078	0.051
**Control- or intervention-period**	1.750	1.222 ─ 2.278	**< 0.001**
**CCI**^**b**^	0.004	-0.168 ─ 0.176	0.961
**Recent hospitalization**	-0.762	-1.503 ─ (-0.021)	**0.044**
**Elective or urgent admission**	-0.532	-1.118 ─ 0.054	0.075
**Fall-risk score on admission**	-0.075	-0.150 ─ 0.000	**0.049**
**Number of DRPs**^**c**^** at discharge**	0.530	0.406 ─ 0.654	**< 0.001**
**Number of drugs on admission**	-0.122	-0.213 ─ (-0.032)	**0.009**

*R*^2^ = 0.507^a^ (Adjusted *R*^2^ = 0.473), F (8,118) = 15.151, *p* < 0.001)

^a^Predictors: (Constant), Age, Control- or intervention-period, CCI, Recent hospitalization, Elective or urgent admission, Fall-risk score on admission, Number of DRPs at discharge, Number of drugs on admission

^b^*CCI* Charlson Comorbidity Index

^c^*DRPs* Drug-Related Problems

### Pharmacist intervention analysis

In the intervention group, the GP was contacted by the CP to discuss DRPs in 56 patients. For 223 DRPs, a total of 141 (median = 2) interventions were suggested in the discharge letter of which 58 (41%) were immediately accepted at discharge and 42 (30%) one month later.

The CI was assessed by a geriatrician and CP for 141 PIs using the CLEO-tool. There was none to slight inter-rater agreement between the two raters in accordance with Kappa Cohen interpretation by Cohen, ĸ_w_=0.185 (95% confidence interval, 0.083 to 0.287), *p* < 0.001. Overall, 15 PIs (11%) had a major CI according to the geriatrician, compared with 0 PIs for the CP. The geriatrician estimated that 83 PIs (59%) had a moderate CI and the CP 63 (45%). A total of 35 PIs (25%) had a minor CI and 8 PIs (5%) had no CI according to the geriatrician, and the CP scored 76 PIs (54%) as having a minor CI and 2 PIs (1%) as having no CI. For example, the drug class presenting the most PIs with a major CI according to the geriatrician was the combination of QT-prolonging drugs (73%) while this was more likely to be considered as having a moderate CI by the CP (17%).

## Discussion

The aim of this study was to investigate the impact of PIs, with regard to reducing DRPs in orthogeriatric patients after discharge. This was done by transmural communication of the proposed PIs at discharge to GPs in order to increase the acceptance rate of the proposed PIs. A multidisciplinary approach was used consisting of the incorporation of a CP in the already existing OG-CM model at the orthopedic ward [7]. The collaboration between the geriatrician and orthopedic surgeons, has proven to improve the quality of care for orthogeriatric patients.

The most common DRPs identified in our study were similar to those in a general geriatric ward [12]. Orthogeriatric patients have a high need for pain relief, which resulted in a number of DRPs involving drugs causing constipation, combinations of anticholinergic drugs, and omission of laxatives in concomitant opioid use. Compared with the study by Kympers et al., the use of PIMs, such as FRIDs, was more frequent. This could be explained by the fact that this study targeted geriatric patients with fall-related injuries.

The multidisciplinary approach in the intervention period was considered successful as it significantly reduced the number of DRPs compared to the control period. Approximately 44% of the existed DRPs were resolved after the PI. This number is lower compared to other studies (58.9–68.3%) [18]. Variations may be explained by differences in the included population, i.e., no age restrictions, with older patients at higher risk for DRP development due to various comorbidities and age-related changes in pharmacokinetics and pharmacodynamics [2]. Additionally, the instruments used to identify DRPs can have an impact. Kympers et al. also used the GheOP^3^s tool to identify DRPs and reported a similar number of DRPs per patient (median = 4) [12], compared to a median of 3 in our study. These numbers are consistent with reported numbers in literature (1.3–3.3 DRPs per patient) [2, 19].

A statistically, significant decrease of 1 DRP per patient was observed in our study in the intervention period compared to the control period. The clinical significance cannot be precisely established but we can make an estimate by looking at which DRPs were resolved one month after discharge in the intervention period (Table 2) and the assessment of the CI of these PIs by the independent geriatrician and CP. For example, we mainly saw a reduction in DRPs such as combination of QT-prolonging drugs, intake of antidepressants > 1 year, intake of PPIs > 8 weeks and combination of drugs leading to increased bleeding risk. All these PIs, for which PIs was proposed, were considered as having a moderate of major CI by the independent geriatrician and CP.

Besides the intervention itself, the number of DRPs at discharge appeared to significantly increase the DRP reduction rate. In other studies, factors such as number of prescribed drugs on admission, CCI and LOS, were shown to be associated with an increase in DRPs [20, 21]. In this study, no association was observed between the increase in DRPs and these determinants. This may be due to differences in the study population, as the other studies mainly focused on patients admitted to a geriatric internal medicine ward. Baseline patient characteristics of both groups in our study were similar, except for the patients’ LOS. About half of the included patients in the intervention group were discharged to a rehabilitation facility, while in the control group only 1 in 3. This could possibly be explained by the longer waiting times for rehabilitation centers due to the COVID pandemic in the intervention period.

In contrast, recent hospitalization and number of drugs on admission, did not result in a decreased DRP rate at one month post-discharge. A possible explanation might be that recently admitted patients were at lower risk of having multiple DRPs, as these DRPs could have been resolved during their previous hospitalization. Remarkably, a higher fall-risk score (≥ 6 higher risk for fall), might be associated with a decrease in DRP reduction rate (Table 3). The timely identification and deprescribing of FRIDs should be of utmost importance in this setting as part of a multifactorial fall-prevention strategy [9]. We hypothesize that GPs may be hesitant to accept interventions regarding these drugs because optimizing drug therapy through deprescribing is very intensive and time-consuming. Motivation of PCPs, knowledge, lack of time for deprescribing, and miscommunication between specialists, PCPs and patients can be either facilitators or barriers of deprescribing [22]. Optimizing the deprescribing process, especially for drugs in which the potential harms outweigh the potential benefits, may improve outcomes for these patients. PCPs involvement is crucial in order to achieve a sustainable DRP reduction [11]. Other professionals such as the CoP can also be included to support the GP with DRP follow-up, thus contributing to improvement of pharmaceutical care. A study with a multifaceted approach and patient-centered and primary care directed intervention, proved to be effective for deprescribing as more than 90% of the older patients agreed to discontinue unnecessary drugs when recommended by their GP [23]. Further research is needed, focusing on these drugs to develop specific interventions that combine explicit and implicit approaches, with patient-centered decision making [24].

Furthermore, the physician acceptance rate of the proposed interventions was high (71%), though lower than reported in other Belgian studies [12, 18]. In contrast, most studies focus on the hospital physician’s acceptance and not the GPs. In the OPERAM trial, an international study also focusing on optimizing drug treatment in geriatric patients, the approach was similarly to ours, except that the GP acceptance rate was set at two months post-discharge (62%) [24]. However, the included patients in this study were not surgical patients and their context might have changed more at two months’ post-discharge compared to one month, resulting in a less likely acceptance of interventions. The personal approach of the GP, with telephone contact at two time points, was considered to add to the success of the PIs.

This study underlines that PIs can have a significant impact on preventing drug-related patient harm. However, the pharmacist-led transmural care program is a time-consuming intervention, and selection of patients at highest risk of DRPs is imperative to make it feasible. In this study, a tool to identify older patients at high risk of DRPs, could be useful to target PIs for patients with high pharmaceutical needs due to factors such as illness severity, co-morbidities, high-risk drugs and polypharmacy. These tools should contribute to more informed discussions between patients and GPs on reducing DRPs, thus breaking down some barriers to deprescribing. Literature on how to properly define these high-risk patients is still scarce [25, 26]. Therefore, more studies are needed on patient selection in order to optimize resources and implement this intensified form of medication review in practice [2].

Regarding CI, 70% of PIs were rated by the geriatrician as having the potential to avoid patient harm while for the CP it was 45%. These results are similar to other findings [27]. Our results showed low agreement raters for the inter-rater reliability which is comparable to results obtained in other studies by Somers et al. (Kappa = 0.15–0.25) and lower than Vo et al. (kw = 0.41) suggesting the difficulty of CI assessment [16, 28]. These differences clearly show the importance of a multidisciplinary approach given the point of view and focus of geriatricians and CPs is different in evaluating the CI of PIs.

The study has potential limitations. First, this study was performed in a single hospital, limiting generalizability. Second, we did not assess the effect of the intervention on clinical outcomes such as readmissions as our sample size would not be large enough to ensure adequate power. Neither did we evaluate other relevant outcomes of resolved DRPs, such as cost savings and QOL, or whether the admission was drug-related. The success rate of a PI may be influenced if the GP is made aware of the fact that the admission was drug-related. Another limitation was that patient findings regarding the pharmacist-led transmural care program were not documented. Only a few studies actively involved the patient, as patient input is of high importance [29]. If patients agree with the proposed PIs, they are more likely to follow through with the changes and the chance of the intervention being successful is higher [30]. In this study, the patient was only actively involved in MR and discharge counseling. Finally, the CI of the PIs should be evaluated by an expert panel in order to provide a more balanced result, integrating all perspectives of the involved care providers.

## Conclusion

To conclude, this study showed that a multifaceted pharmacist-led intervention with a primary care directed approach, proved to be effective in order to resolve DRPs and to ensure continuity of care after discharge. In future studies, the patient and CoP should be actively involved to further reduce DRPs. It is important to aim for significantly large sample sizes in order to assess the effectiveness of the intervention on other relevant clinical outcomes such as readmissions.

## Data Availability

The data generated or analyzed during this study are presented in this article and for further enquiries can be directed to the corresponding author.

## Abbreviations

CP: Clinical Pharmacist
GP: General Practitioner
DRPs: Drug Related Problems
CLEO: Clinical, Economic and Organizational
PCNE: Pharmaceutical Care Network of Europe
QOL: Quality Of Life
OG-CM: Orthogeriatric Co-Management
MR: Medication Reconciliation
FRIDs: Fall-Risk Increasing Drugs
PCPs: Primary Care Providers
PIs: Pharmaceutical Interventions
LOS: Length Of Stay
CoP: Community Pharmacist
GheOP^3^S: Ghent Older People’s Prescriptions community Pharmacy Screening
CCI: Charlson Comorbidity Index
CI: Clinical Impact
SD: Standard Deviation
IQR: Interquartile Range
κ_w_: Weighted Cohen’s Kappa
PIMs: Potentially Inappropriate Medication
PPIs: Proton-Pump Inhibitors
NSAIDs: Non-Steroidal Anti-Inflammatory Drugs
RAAS: Renin-Angiotensin-Aldosteronesystem
